# Wilms’ Tumor 1-Associated Protein Contributes to Chemo-Resistance to Cisplatin Through the Wnt/β-Catenin Pathway in Endometrial Cancer

**DOI:** 10.3389/fonc.2021.598344

**Published:** 2021-02-16

**Authors:** Wenli Xie, Naifu Liu, Xiangyu Wang, Ling Wei, Wenyan Xie, Xiugui Sheng

**Affiliations:** ^1^ School of Medicine, Shandong University, Jinan, China; ^2^ Department of Gynecologic Oncology, Shandong Cancer Hospital and Institute, Shandong First Medical University and Shandong Academy of Medical Sciences, Jinan, China; ^3^ Shandong Provincial Key Laboratory of Radiation Oncology, Shandong Cancer Hospital and Institute, Shandong First Medical University and Shandong Academy of Medical Sciences, Jinan, China; ^4^ Department of Clinical Laboratory, Shandong Provincial Qianfoshan Hospital, Shandong University, Jinan, China; ^5^ National Cancer Center, National Clinical Research Center for Cancer and Cancer Hospital & Shenzhen Hospital, Chinese Academy of Medical Sciences and Peking Union Medical College, Shenzhen, China

**Keywords:** Wilms’ tumor 1-associated protein, endometrial cancer, cisplatin, chemo-sensitivity, Wnt

## Abstract

**Background:**

Cisplatin remains the mainstay of endometrial cancer (EC) chemotherapy. Wilms’ tumor 1-associated protein (WTAP), playing a critical role in transcriptional and post-transcriptional regulation, has been reported as an oncogene, and its expression is elevated in multiple types of human tumors. Recent evidence has shown that the increased expression of WTAP is also closely related to chemo-resistance. However, its specific role in the susceptibility of human EC cells to cisplatin remains largely unexplored.

**Methods:**

WTAP over-expression and WTAP depletion cell lines as well as their corresponding controls were constructed by transfection with lentivirus. Western blotting analysis and quantitative real-time polymerase chain reaction (qRT-PCR) were employed to detect the expression of WTAP. Cell proliferation assay, colony formation assay, cell cycle assay, and apoptosis analysis were adopted to evaluate the effect of WTAP on the chemo-sensitivity of EC cells to cisplatin as well as its underlying mechanism. Immunofluorescence staining was used to assess the translocation of β-catenin. Moreover, a subcutaneous xenograft tumor model was established to assess the effect of WTAP on tumor growth after cisplatin treatment.

**Results:**

Depletion of WTAP in RL95-2 cells significantly enhanced the chemo-susceptibility of cells to cisplatin and increased the cell apoptosis, while WTAP over-expression in ARK-2 cells exhibited the opposite effects. Additionally, WTAP depletion significantly suppressed xenograft-tumor growth and enhanced sensitivity and apoptosis of tumor cells *in vivo*. Mechanistic analysis exhibited that WTAP over-expression facilitated the cytoplasm-to-nucleus translocation of β-catenin and enhanced the GSK3β phosphorylation at Ser9, while WTAP depletion revealed the opposite results, indicating that WTAP rendered chemo-resistance of EC cells to cisplatin by promoting the Wnt/β-catenin pathway.

**Conclusions:**

WTAP might promote the chemo-resistance of EC cells to cisplatin through activating the Wnt/β-catenin pathway. Collectively, our findings offered novel insights into EC treatment.

## Introduction

Endometrial cancer (EC) is a common gynecological malignancy in the United States, with more than 65,620 newly diagnosed cases every year (1). Surgery is the typical therapeutic strategy for early-stage EC, while those with advanced and/or recurrent EC are mainly subjected to systemic chemotherapy in combination with radiotherapy (2). Unfortunately, the curative effect of most current chemotherapeutic drugs, including cisplatin, the first-line chemotherapy regimen for EC treatment, is less evident for advanced EC patients, and cisplatin resistance remains a common challenge in EC treatment. However, new therapeutic measures are being studied, and additional therapies that can improve the outcomes of existing treatment may immediately benefit the patients with advanced or recurrent EC.

Wilms’ tumor 1-associated protein (WTAP), a nuclear protein, can specifically interact with WT1 as its name implies (3). Besides its essential physiological processes in cell cycle regulation (4), mRNA stabilization (5), RNA alternative splicing (6), m6A methylation (7), and eye development (8), WTAP has also been demonstrated to act as an oncogene in the tumorigenesis of malignant cancers, such as renal cell carcinoma (RCC), glioma, colorectal cancer (CRC), pancreatic ductal adenocarcinoma (PDAC), cholangiocarcinoma (CCA), ovarian cancer, bladder cancer, and acute myelogenous leukemia (AML) (9–17).

Furthermore, recent evidence has revealed that chemo-resistance is triggered by increased expression of WTAP in PDAC and AML following etoposide- and gemcitabine-based chemotherapy, respectively (12, 18). WTAP depletion alone does not induce cell apoptosis in AML, while the extent of apoptosis is dramatically enhanced when combined with etoposide (12). In PDAC, WTAP promotes chemo-resistance to gemcitabine by stabilizing Fak mRNA through activating Fak signaling pathways. Nevertheless, the functions of WTAP in the tumorigenesis of EC, including its role in the chemo-sensitivity of human EC cells to cisplatin, have not been fully documented so far.

In our current work, we provided the first evidence that increased expression of WTAP enhanced the chemo-resistance to cisplatin by decreasing the apoptosis of tumor cells and reducing the proportion of EC cells in the G2/M phase, while depletion of WTAP led to an opposite result. The findings were further corroborated in nude mouse xenograft models, in which WTAP over-expression could facilitate the chemo-resistance of EC cells to cisplatin. Further investigation indicated that the Wnt/β-catenin pathway participated in the chemo-resistance effect of WTAP. Collectively, these findings supported WTAP as a promising therapeutic target for EC due to its potential contribution to the chemo-sensitivity of EC cells to cisplatin.

## Materials and Methods

### Cell Lines and Culture Conditions

Human EC-derived cell lines RL95-2, Ishikawa, HEC-1B, and ARK-2 were obtained from the American Type Culture Collection (ATCC, Manassas, VA, USA). Ishikawa, HEC-1B, and ARK-2 cells were maintained in Dulbecco’s modified Eagle’s medium (DMEM, ThermoFisher, Carlsbad, CA, USA) supplemented with 10% fetal bovine serum (FBS, ThermoFisher, USA) and 1% penicillin/streptomycin (Gibco, USA) at 37°C in a humidified atmosphere containing 5% CO_2_, whereas RL95-2 cells were grown in DMEM/F-12 (HyClone, Biological Industries, Israel) supplemented with 10% FBS (Invitrogen, USA), 1% antibiotics, and 5 µg/ml insulin (Procell, Wuhan, China) under above-mentioned conditions.

### Reagents and Antibodies

Cisplatin (MedChem Express, USA) was dissolved in H_2_O to make a stock solution at the concentration of 1 mg/ml (3.33 mM) and stored in single-use tubes at −80°C. Anti-WTAP, anti-CDK1, and anti-BAX antibodies were supplied by Abcam (USA). Anti-Mcl-1, anti-Wee1, anti-β-catenin, anti-GSK-3β, anti-GSK-3β pSer9, anti-H2A, and anti-GAPDH antibodies were provided by Cell Signaling Technology Inc. (China). Anti-PARP antibody, anti-ki67 antibody, anti-β-actin antibody, and goat anti-rabbit IgG were obtained from ProteinTech Group Inc. (USA).

### RNA Extraction and Quantitative Real-Time Polymerase Chain Reaction

Total RNA was isolated from cells using Trizol reagent (Invitrogen, USA) according to the manufacturer’s instructions. The purified RNA was reversely transcribed into cDNA using the HiFiScriptcDNA Synthesis Kit (CWBIO, CW2569, China) according to the manufacturer’s instructions. qRT-PCR was conducted on a LightCycler 480 qPCR system (Roche Diagnostics, Germany) using UltraSYBR Mixture (CWBIO, CW0956, China) following the manufacturer’s instructions. The cells with the lowest expression of WTAP at the mRNA level were used as the calibrator sample when evaluating the relative expression of WTAP at the mRNA level among four EC cell lines. Fold changes of target genes at the mRNA level were calculated using the 2^-ΔΔCt^ method and normalized based on β-actin.

The primer sequences used were as follows:

WTAP forward: 5′-CTGACAAACGGACCAAGTAATG- 3′

WTAP reverse: 5′-AAAGTCATCTTCGGTTGTGTTG- 3′

c-Myc forward: 5′**-**CTCCTACGTTGCGGTCACAC**-** 3′

c-Myc reverse: 5′**-**TGATGAAGGTCTCGTCGTCC**-** 3′

Survivin forward: 5′**-**TTTCTCAAGGACCACCGCATC**-** 3′

Survivin reverse: 5′**-**CAAGTCTGGCTCGTTCTCAG**-** 3′

Bcl-xl forward: 5′**-**GGCAGCAGTAAAGCAAGCG**-** 3′

Bcl-xl reverse: 5′**-**GCTCTGATATGCTGTCCCTGG-3′

β-actin forward: 5′-GGCGGCACCATGTACCCT- 3′

β-actin reverse: 5′-AGGGGCCGGACTCGTCATACT- 3′

### Cytoplasmic and Nuclear Extraction

The extraction of cytoplasmic and nuclear proteins was carried out using the Nuclear and Cytoplasmic Protein Extraction Kit (Beyotime, China) according to the manufacturer’s instructions. Briefly, cells were washed, harvested, and resuspended with cytoplasmic protein extraction buffer A supplemented with PMSF. Following vortex for 5 s, the obtained cell lysates were incubated on ice for 15 min. Subsequently, cytoplasmic protein extraction buffer B was added, followed by incubation on ice for 5 s. After centrifuged at 12,000 g for 5 min at 4°C, the pellets were re-suspended in nuclear extraction buffer supplemented with PMSF and vigorously shaken at 4°C for 30 min. After centrifugation at 12,000 g for 10 min, the resulting supernatants containing the nuclear extracts were obtained.

### Protein Extraction and Western Blotting Analysis

Western blotting analysis was performed as described previously (18). Total proteins were isolated using the RIPA buffer [50 mM Tris pH 8.0, 1% NP-40, 150 mM NaCl, 0.1% sodium dodecylsulfate (SDS) and 1 mM ethylenediaminetetraacetic acid (EDTA)] containing 0.5 mM phenylmethanesulfonylfluoride (Beyotime, China). The protein concentration was determined using the BCA protein assay kit (Beyotime, China) following the manufacturer’s instructions. Equal amounts of proteins were subjected to sodium dodecyl sulphate-polyacrylamide gel electrophoresis (SDS-PAGE) on 10% gels and then electro-transferred onto polyvinylidene difluoride (PVDF) membranes (Millipore, USA). Subsequently, the membranes were blocked with 5% nonfat dry milk (Bio-rad, USA) in Tris-buffered saline (TBS, 150 mM NaCl, 50 mM Tris-HCl, pH 7.5) containing 0.05% Tween-20 for 1 h, followed by incubation with primary antibodies against WTAP (1:1,000), CDK1 (1:4,000), BAX (1:2,000), Mcl-1 (1:1,000), Wee1 (1:1,000), GSK-3β (1:1,000), GSK-3β pSer9 (1:1,000), H2A (1:1,000), GAPDH (1:1,000), or β-actin (1:1,000). Next, the membranes were incubated with horseradish peroxidase (HRP)-conjugated secondary antibody (1:2,000) at room temperature for 1 h. Immunoreactive bands were visualized using enhanced chemiluminescence (ECL) detection reagents (ProteinTech, USA).

### Transfection

Lentiviral vectors carrying human WTAP cDNA (WTAP over-expression) and WTAP shRNA (WTAP depletion) were prepared and constructed by Gene-Pharma (Shanghai, China). Cells were seeded into 6-well plates and maintained for 24 h. Once the cell confluence of 40% was achieved, cells were subjected to infection with WTAP depletion lentivirus (termed as shWTAP), scramble control lentivirus (termed as shNC), WTAP over-expression lentivirus (termed as WTAP-OE), and negative control lentivirus (termed as WTAP-NC). Transfected RL95-2 and ARK-2 cells were subjected to puromycin selection at concentrations of 1 and 5 μg/ml for 2 weeks, respectively. Transfection efficiency was confirmed by Western blotting analysis and qRT-PCR. The expression of WTAP at the mRNA level in cells transfected with shNC or WTAP-NC lentivirus was treated as the touchstone for statistical analysis when evaluating the transfection efficiency using qRT-PCR data.

### Cell Viability Assay

For cell viability analysis, EC cells were seeded into 96-well plates at a density of 1×10^4^ to 1.5×10^4^ cells/well, followed by incubation for 24 h. The attached cells were exposed to cisplatin at various concentrations for 48 h. The dose of cisplatin was 0, 0.125, 0.25, 0.5, 1, 2, and 4 μg/ml in RL95-2 cells, while it was 0, 0.25, 0.5, 1, 2, 4, 8, and 16 μg/ml in ARK-2 cells. The number of viable cells was determined using the cell counting kit-8 (CCK-8, MedChem Express, USA) assay following the manufacturer’s instructions. The experiment at each concentration was carried out in triplicate.

### Colony Formation Assay

Colony formation assay was carried out as previously described (19). Briefly, 150–500 cells were plated into each well of a 6-well plate and exposed to cisplatin at different doses. Drug intervention was terminated after 48 h, and cells were further cultured for 2 weeks under normal conditions. Cells were fixed with 4% paraformaldehyde (PFA) for 20 min and stained with 0.1% crystal violet, and then colonies composed of > 50 cells were identified. The colony formation efficiency (CFE) was determined using the equation as follows: CFE = clones/cell numbers × 100%. The CFE without cisplatin treatment was used as the calibrator when evaluating the surviving fraction at each concentration: surviving fraction = CFE with cisplatin treatment/CFE without cisplatin treatment × 100%.

### Cell Cycle Assay

Cell cycle analysis was performed as described previously (19). Cells were harvested, washed with PBS, and fixed in 70% cold ethanol for 24 h. After staining in 500 μl propidium iodide (PI) working solution (20 μg/ml PI, 0.1% Triton X-100, 200 μg/ml DNase-free RNase A) in the dark at room temperature for 30 min, DNA content was determined using a FACS Calibur instrument (Becton Dickinson, USA), and the cell cycle distribution was further analyzed by ModiFit software (Topsham, ME, USA).

### Terminal Deoxynucleotidyl Transferase-Mediated dUTP Nick End*-*Labeling Assay

Briefly, cells were fixed with 4% PFA for 1 h, while the xenograft tumor tissues of nude mice were fixed with formalin and embedded in paraffin. One-step TUNEL *in situ* cell apoptosis detection kit (Keygen, China) was used to determine cell apoptosis by specific staining following the manufacturer’s instructions. The numbers of TUNEL-positive cells and total cells were evaluated using a confocal laser microscope (LSM800, Carl Zeiss, Germany). The proportion of apoptotic cells was determined as apoptotic cells/total cells ×100%.

### Immunofluorescence Staining

IF analysis was performed as described previously (19). Briefly, cells were seeded onto chamber slides, then fixed with 4% PFA for 10 min, permeabilized for 15 min with 0.1% Triton X-100, and blocked with 5% bovine serum albumin (BSA) for 1 h. Subsequently, cells were incubated with anti-β-catenin antibody (1:100) at room temperature for another 1 h. Then the slides were washed with PBS, followed by incubation with Coralite 594-conjugated secondary antibodies (1:500, ProteinTech, USA) for 1 h. Finally, cells were stained with 4′, 6-diamidino-2-phenylindole (DAPI) (Beyotime, China) for 3 min. Images were captured with the confocal laser microscope.

### BALB/c Nude Mice Xenograft Model

All animal studies complied with the Institutional Animal Care and Use Committee of Shandong Cancer Hospital and Institute (SDTHEC-2020005005). Female BALB/c nude mice (4–6 weeks old) were obtained from Beijing HFK Bioscience Co., Ltd. (Beijing, China). Transfected RL95-2 or ARK-2 cells were suspended in PBS at a density of 2 × 10^7^ cells/ml, and 200 μl cell suspension was subcutaneously inoculated into the right flank of each nude mouse (n = 10). When the tumor volume reached approximately 150 mm^3^, mice were randomly divided into four groups (n = 5), and intraperitoneally administered with cisplatin. The xenograft models constructed with transfected RL95-2 and ARK-2 cells were administered with cisplatin at concentrations of 3 mg/kg and 5 mg/kg, respectively. Cisplatin was injected every 4 days for 16 days. Tumor size was determined every 3 days using a caliper and calculated using the formula as follows: tumor volume = length×width^2^ × 0.5. After treatment completion, animals were sacrificed, and tumors were excised and weighed for immunohistochemical (IHC) assay.

### Histological and IHC Assays

The xenograft tumor tissues were fixed in formalin, embedded in paraffin, sectioned, and stained with hematoxylin-eosin (H&E) following the documented procedures (19). IHC analysis was performed as previously described (20). Briefly, the sections were dewaxed and rehydrated, followed by citrate antigen retrieval. Subsequently, the sections were blocked by endogenous peroxidase and then incubated with primary antibodies against WTAP (1:100) and ki67 (1:16,000) at 4°C overnight. Next, sections were subjected to incubation with HRP-conjugated secondary antibody and DAB peroxidase substrate. The slides were analyzed with light microscopy (Olympus, Japan).

### Bioinformatics Analysis

The gene set enrichment analysis (GSEA) was carried out following a standard protocol as previously described (21, 22). To investigate the potential pathways contributing to the chemo-resistance effect of WTAP, the GSEA software 3.0 was used to assess the GEO database (GEO/GSE126346, GEO/GSE17025), which is the transcription profiling by an array of EC patients based on a previously established approach. The random sample permutation number was set as 1,000 for each analysis. GSEA reveals statistical differences (false discovery rate (FDR) < 0.25 and normalized p-value <0.05) in enrichment of MSigDB Collection (h.all.v6.2.symbols.gmt) for a certain gene set. The results were visualized with enrichment maps generated using Bioconductor (http://bioconductor.org/) and R v3.6.0 software (R Foundation, Vienna, Austria).

### Statistical Analysis

Statistical analysis was performed using GraphPad Prism version 5 (GraphPad Software, USA). Statistical significance was assessed with data from at least three independent experiments and the data were expressed as means ± SD. The statistical significances between two groups were determined using Student’s t-test. P values < 0.05 were considered statistically significant.

## Results

### RL95-2 and ARK-2 Are Chosen for More Investigation

**Figure 1:**
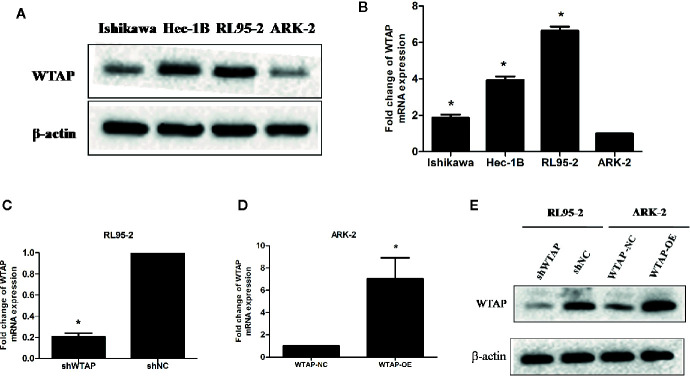
RL95-2 and ARK-2 were selected as target cells and subjected to various treatments. **(A, B)** The expression of WTAP at the protein and mRNA levels in four EC cell lines. **(C–E)** The efficiency of WTAP depletion and WTAP over-expression was validated by qRT-PCR and Western blotting analysis. The data are presented as the mean ± SD from three independent experiments (*P < 0.05). WTAP, WT1-associated protein; shWTAP, WTAP depletion; shNC, scramble control; WTAP-OE, WTAP over-expression; WTAP-NC, WTAP negative control.

The expression of WTAP in four different EC cell lines (RL95-2, Ishikawa, Hec-1B, and ARK-2) was assessed using Western blotting analysis and qRT-PCR. **Figure 1A** illustrates that the highest WTAP expression was observed in RL95-2 cells, whereas its lowest expression was detected in ARK-2 cells. qRT-PCR showed that the expression of WTAP at the mRNA level was consistent with its protein expression (**Figure 1B**). As a result, RL95-2 and ARK-2 were selected for further experiments. According to the methods described in the above section, stable transductions of WTAP depletion and over-expression were achieved using RL95-2 and ARK-2, respectively. The efficiency of WTAP depletion and WTAP over-expression was further validated by qRT-PCR (**Figures 1C, D**) and Western blotting analysis (**Figure 1E**).

### WTAP Depletion Increases Chemo-Sensitivity and Suppresses Colony Formation in Human EC Cells

**Figure 2:**
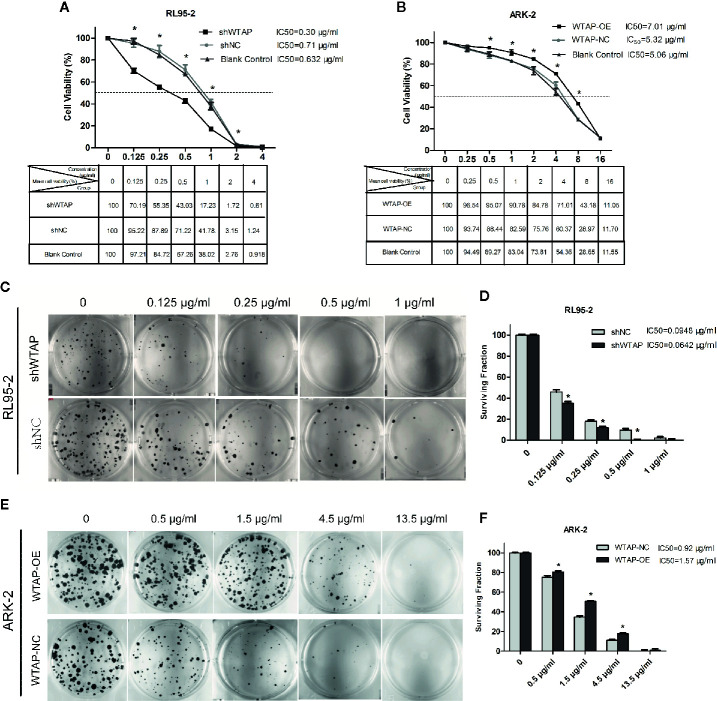
Effects of WTAP on the proliferation and colony formation of EC cells in response to cisplatin. **(A)** Viability rates of WTAP-depleted RL95–2 cells after exposed to cisplatin at various concentrations (0–4 μg/ml) for 48 h. Cells transfected with shNC and normal RL95–2 cells served as controls. **(B)** Viability rates of WTAP-overexpressing ARK–2 cells after exposed to cisplatin at various concentrations (0–16 μg/ml). Cells transfected with WTAP-NC and normal ARK–2 cells served as controls. **(C, D)** Both the WTAP-depleted RL95–2 cells and control cells were treated with cisplatin (0.125, 0.25, 0.5, and 1 μg/ml) and subjected to cell colony formation assay. Cells without cisplatin treatment served as the control. **(E, F)** Both WTAP-overexpressing ARK–2 cells and corresponding control cells were exposed to cisplatin at various concentrations (0.5, 1.5, 4.5, and 13.5 μg/ml) and subjected to cell colony formation assay. Cells without cisplatin treatment served as the control. *P < 0.05. IC50: the 50% maximal inhibitive concentration. shWTAP, WTAP depletion; shNC, scramble control; WTAP-OE, WTAP over-expression; WTAP-NC, WTAP negative control.

Cell toxicity test was performed to evaluate the effect of WTAP on cisplatin chemo-sensitivity in EC cells. **Figures 2A, B** reveal that depletion of WTAP enhanced the inhibitory activity of cisplatin in RL95-2 cells. The 50% maximal inhibitive concentration (IC50) of cisplatin in cells transfected with shWTAP (0.30 µg/ml) was lower compared with the control group (0.71 µg/ml) and normal RL95-2 cells (0.632 µg/ml). Conversely, over-expression of WTAP compromised the cisplatin inhibitory activity in ARK-2 cells. The IC50 of cisplatin in the WTAP-OE group (7.01 µg/ml) was greater compared with the WTAP-NC group (5.32 µg/ml) and normal ARK-2 cells (5.06 µg/ml). Further cell colony formation assay validated the above-mentioned findings. Once exposed to cisplatin, RL95-2 cells infected by WTAP depletion lentivirus (IC50 = 0.0642 μg/ml) had a lower colony formation rate compared with the control cell lines (IC50 = 0.0948 μg/ml), whereas the proportion of colony formation in ARK-2 cells infected by WTAP-OE lentivirus (IC50 = 1.57 μg/ml) was dramatically higher compared with the ARK-2 cells infected by WTAP-NC lentivirus (IC50 = 0.92 μg/ml) (**Figures 2C–F**). Taken together, these data suggested that WTAP contributed to the chemo-resistance of EC cells to cisplatin *in vitro*.

### WTAP Depletion Enhances the Cisplatin-Induced Apoptosis and Induces Cell Cycle Arrest to Cisplatin *In Vitro*

**Figure 3:**
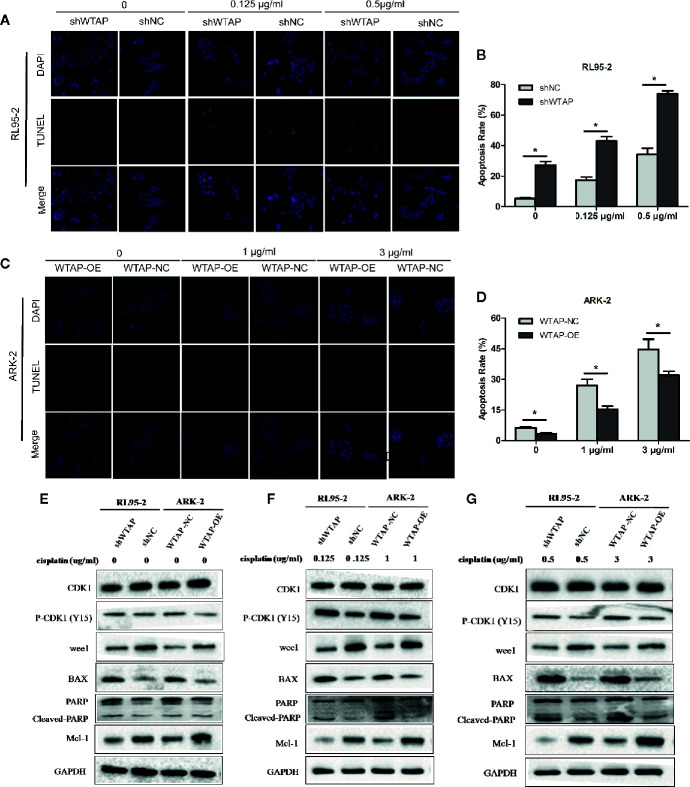
WTAP affects cisplatin-induced apoptosis of human EC cells. The apoptosis level of WTAP-depleted RL95-2 cells **(A, B)** and WTAP-overexpressing ARK-2 cells **(C, D)** at 48 h post cisplatin treatment was evaluated by TUNEL assays. **(E–G)** Western blotting analysis of G2/M phase-regulated proteins [CDK1, P-CDK1 (Tyr 15), Wee1] and apoptosis-associated proteins (BAX, cleaved-PARP, and Mcl-1) when cells were treated in the absence or presence of cisplatin. **(E)** Either of the two cells did not receive cisplatin treatment; **(F)** WTAP-depleted RL95-2 cells and WTAP-overexpressing ARK-2 cells were exposed to 0.125 and 1 μg/ml cisplatin, respectively. **(F)** WTAP-depleted RL95-2 cells and WTAP-overexpressing ARK-2 cells were exposed to 0.5 and 3 μg/ml cisplatin, respectively. GAPDH was used as a loading control. *P < 0.05. shWTAP, WTAP depletion; shNC, scramble control; WTAP-OE, WTAP over-expression; WTAP-NC, WTAP negative control.

To explore the impact of WTAP on the sensitivity of human EC cells to cisplatin, we performed a TUNEL assay to assess the apoptosis level of EC cells after exposed to cisplatin (0.125 μg/ml or 0.5 μg/ml for RL95-2 cells; 1 μg/ml or 3 μg/ml for ARK-2 cells) for 48 h. **Figures 3A–D** illustrate that decreased expression of WTAP enhanced the apoptosis of RL95-2 cells compared with the control group at both tested concentrations (P < 0.05). Meanwhile, the apoptosis level of ARK-2 cells over-expressing WTAP was obviously lower compared with the control group (P < 0.05). Further Western blotting analysis confirmed the above-mentioned results (**Figures 3E–G**). After cisplatin treatment for 48 h, the expressions of pro-apoptotic proteins BAX and cleaved PARP were higher in the shWTAP group compared with the shNC group in RL95-2 cells, while the expression of Mcl-1, an anti-apoptotic protein, was lower in WTAP-depleted cells. Conversely, the expressions of BAX and cleaved PARP were lower in WTAP-overexpressing ARK-2 cells compared with the control cells, while the expression of Mcl-1 was higher.

**Figure 4:**
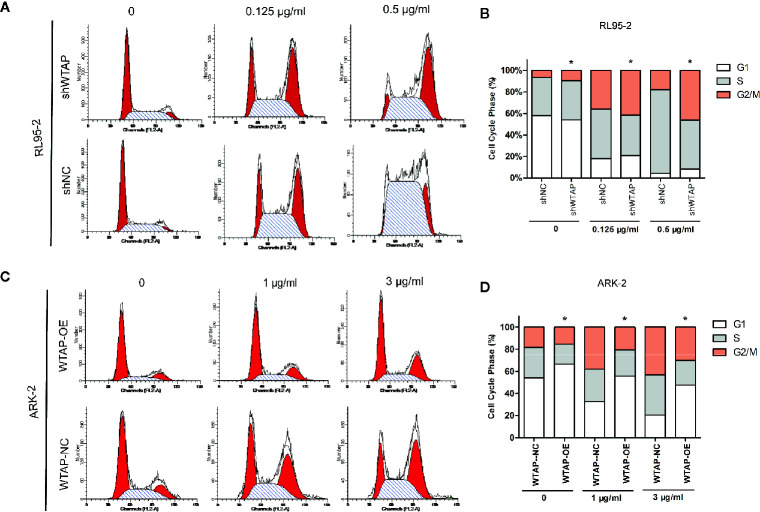
WTAP affects cisplatin-induced cell cycle progression of human EC cells. Distribution of the cell cycle in WTAP-depleted RL95–2 cells **(A, B)** and WTAP-overexpressing ARK–2 cells **(C, D)** compared with the corresponding controls in response to cisplatin treatment. There was an obvious G2/M phase arrest in WTAP-depleted RL95–2 cells and a significant increase of G2/M cells in WTAP-overexpressing ARK–2 cells after cisplatin treatment. *P < 0.05. shWTAP, WTAP depletion; shNC, scramble control; WTAP-OE, WTAP over-expression; WTAP-NC, WTAP negative control.

Besides, flow cytometry showed that the proportion of cells in the G2/M phase (P < 0.05) was increased after cisplatin treatment for 48 h in WTAP-depleted RL95-2 cells, whereas WTAP-overexpressing ARK-2 cells exhibited the opposite effects, showing a reduced proportion of cells in the G2/M phase (**Figures 4A–D**). Moreover, the expression of Wee1 kinase, which acts as a critical G2/M phase-regulated protein (23), was reduced, and the expression of P-CDK1 (Tyr 15), the phosphorylation of which by Wee1 can prevent the cyclin B-CDK1 complex from driving cells into mitosis, was increased in WTAP-depleted RL95-2 cells, resulting in G2 phase arrest (24, 25), while their expressions were changed reversely in WTAP-overexpressing ARK-2 cells (**Figures 3E–G**). However, WTAP did not affect the expression of total CDK1. These results revealed that WTAP promoted chemo-resistance of EC cells to cisplatin *via* facilitating the cell apoptosis and inducing the cell cycle G2/M phase arrest.

### WTAP Promotes Chemo-Resistance to Cisplatin *via* Activating the Wnt/β-Catenin Pathway

**Figure 5:**
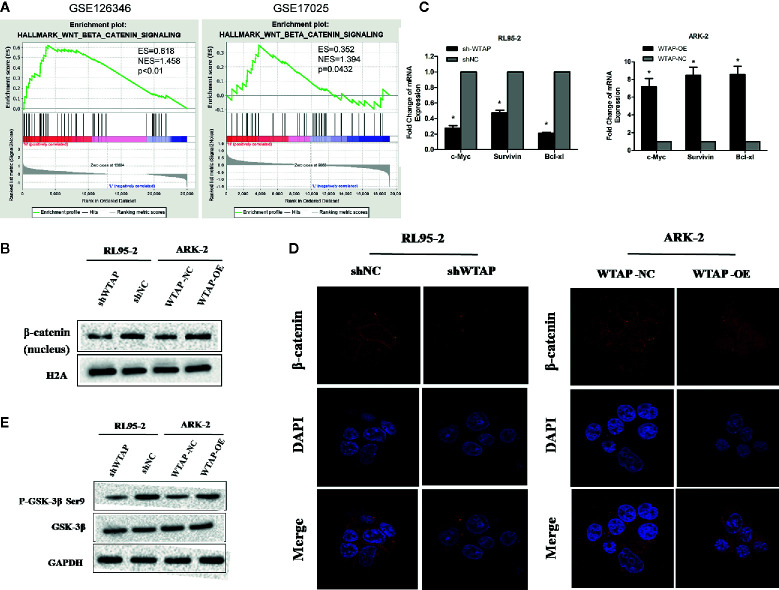
WTAP may promote chemo-resistance to cisplatin *via* activating the Wnt/β-catenin pathway. **(A)** GSEA associated levels of WTAP with Wnt/β-catenin pathway genes on publicly available EC patient gene expression profiles. **(B)** Western blotting analysis of β-catenin in the nuclear extracts of the indicated cells. **(C)** Results of real-time PCR analysis of apoptosis-assosiated genes downstream the wnt/β-catenin pathway in the indicated cells. **(D)** Representative IF images of subcellular β-catenin localization in the indicated cells. **(E)** Western blotting analysis of the GSK-3β and P-GSK-3β (Ser9) in the indicated cells. Both GAPDH and H2A were used as loading controls. *P < 0.05. shWTAP, WTAP depletion; shNC, scramble control; WTAP-OE, WTAP over-expression; WTAP-NC, WTAP negative control.

To investigate the mechanism underlying the WTAP-induced chemo-resistance to cisplatin of EC cells, we conducted GSEA using the GEO database (NCBI/GEO/GSE126346 and NCBI/GEO/GSE17025) *via* GSEA software. We found that WTAP was notably correlated with the Wnt/β-catenin signaling pathway (**Figure 5A**). Therefore, we examined the expression of β-catenin using Western blotting analysis in nuclear extracts from WTAP-depleted cells and WTAP-overexpressing cells. The expression of nuclear β-catenin was up-regulated in WTAP-overexpressing ARK-2 cells and down-regulated in WTAP-depleted RL95-2 cells (**Figure 5B**). Moreover, the expressions of downstream target genes at the mRNA level involved in the Wnt/β-catenin pathway that are correlated with apoptosis were quantified by real-time PCR (**Figure 5C**). Results indicated that the expressions of c-Myc, Survivin, and Bcl-xl were all significantly decreased in WTAP-depleted RL95-2 cells compared with the corresponding control cells. We also found that the expressions of c-Myc, Survivin, and Bcl-xl were significantly increased in the WTAP-overexpressing ARK-2 cells compared with the corresponding control cells. Additionally, IF staining revealed increased cytoplasm-to-nucleus translocation of β-catenin in WTAP-overexpressing ARK-2 cells, while such finding was not observed in WTAP-depleted RL95-2 cells (**Figure 5D**), suggesting that WTAP activated the Wnt/β-catenin pathway by contributing to β-catenin translocation.

Glycogen synthase kinase 3β (GSK3β) is a critical regulatory molecule of the Wnt/β-catenin pathway. The complex formed by GSK3β, adenomatous polyposis coli (APC), and Axin is destroyed once GSK3β is phosphorylated at residue Ser9, which is the mechanism underlying the nuclear translocation of β-catenin. In the present study, we demonstrated that the phosphorylation of GSK-3β (Ser9) was dramatically increased by WTAP over-expression in ARK-2 cells and significantly reduced by WTAP depletion in RL95-2 cells, whereas the expression of GSK-3β was not obviously changed in either of the two cells (**Figure 5E**). Therefore, WTAP activated the Wnt/β-catenin pathway by promoting the GSK3β phosphorylation at Ser9 and the subsequent nuclear translocation of β-catenin.

### Depletion of WTAP Elevates the Sensitivity of Cancer Cells to Cisplatin *In Vivo*

**Figure 6:**
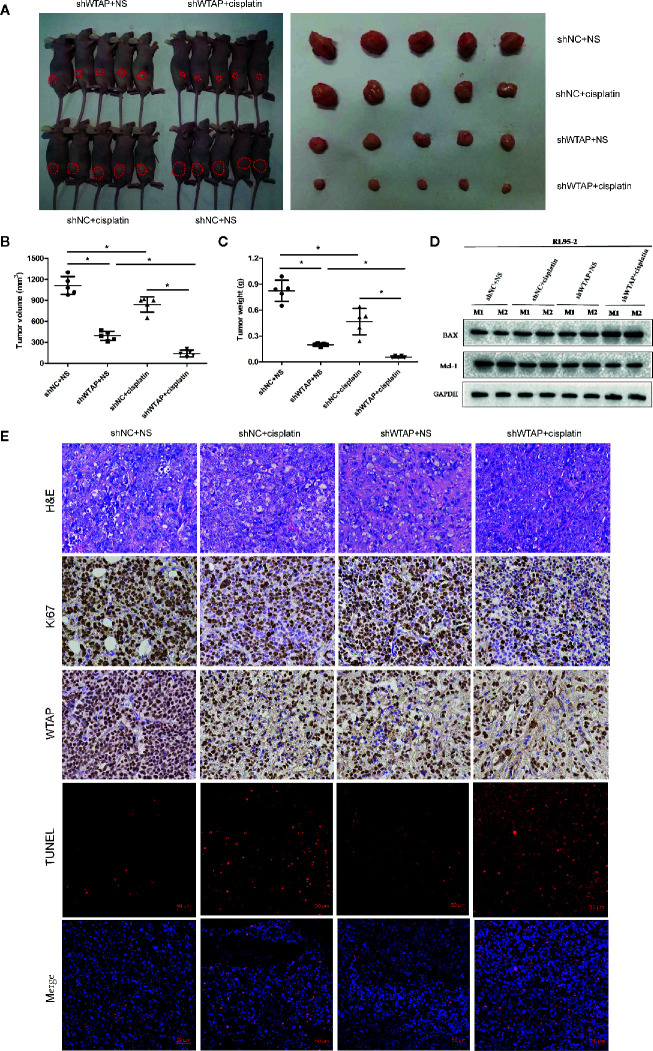
Depletion of WTAP promotes the chemo-sensitivity of EC cells to cisplatin *in vivo*. The mice were inoculated with shWTAP or shNC transfected RL95-2 cells and given NS (control group) or cisplatin (treatment group). **(A)** Mice in the shWTAP plus cisplatin group formed smaller tumors compared with the shNC plus cisplatin group or shWTAP plus NS group. **(B, C)** Comparison of the tumor volume **(B)** and tumor weight **(C)** in mice of each group. **(D)** Western blotting analysis of two mice from each group for BAX and Mcl-1. **(E)** Paraffin-embedded tumor sections were subjected to H&E staining and IHC staining *via* anti-WTAP, and anti-Ki67 antibodies; TUNEL assays were detected in four groups. GAPDH was used as a loading control. NS, normal saline, *P < 0.05. shWTAP, WTAP depletion; shNC, scramble control.

**Figure 7:**
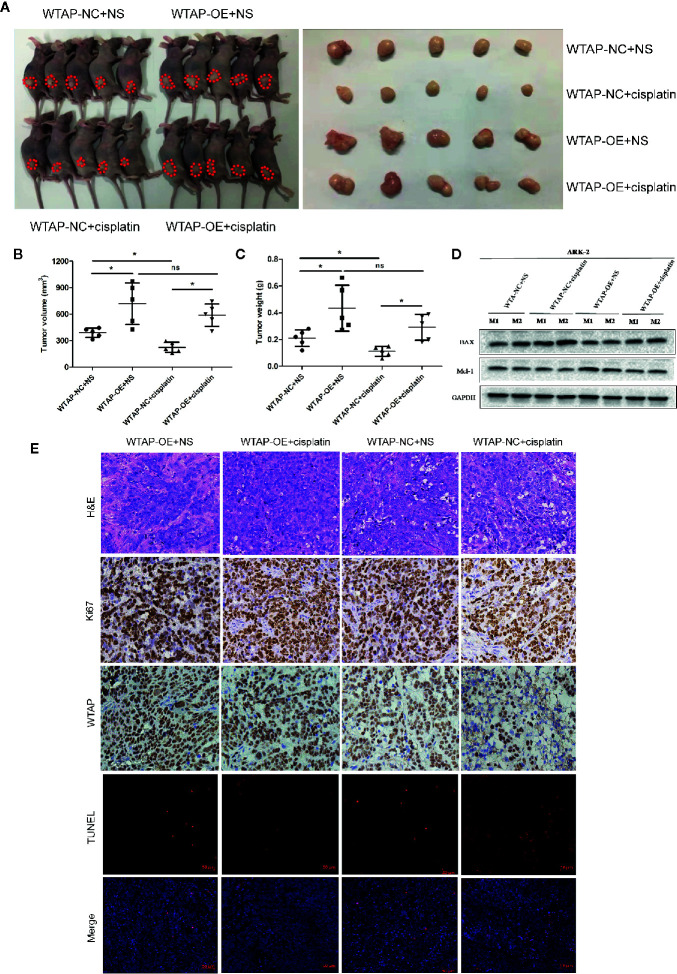
WTAP over-expression contributes to chemo-resistance to cisplatin *in vivo*. The mice were inoculated with WTAP-overexpressing cells or the corresponding control ARK-2 cells and given NS (control group) or cisplatin (treatment group). **(A)** WTAP-OE plus cisplatin group demonstrated significantly higher tumor weight and tumor volume compared with the WTAP-NC plus cisplatin group, while there was no significant difference when compared with the WTAP-OE plus NS group. **(B, C)** Comparison of the tumor volume **(B)** and tumor weight **(C)** in mice of each group. **(D)** Western blotting analysis on tumors from two mice from each group for BAX and Mcl-1. **(E)** Paraffin-embedded tumor sections were subjected to H&E staining and IHC staining *via* anti-WTAP and anti-ki67 antibodies; TUNEL assays were detected in four groups. GAPDH was used as a loading control. NS, normal saline; ns, no significance; *P < 0.05. WTAP-OE, WTAP over-expression; WTAP-NC, WTAP negative control.

In the present study, the subcutaneous xenograft tumor model was established to further explore the impacts of WTAP on tumor growth in response to cisplatin *in vivo*. Expectedly, the tumor volume and tumor weight in the shWTAP plus cisplatin group were significantly inhibited compared with the other groups (**Figures 6A–C**). In contrast, the tumor weight and tumor volume in the WTAP-OE plus cisplatin group were significantly higher compared with the WTAP-NC plus cisplatin group, while there was only a decreasing trend compared with the WTAP-OE plus NS (normal saline) group (**Figures 7A–C**). Moreover, both tumor volume and tumor weight in the shWTAP group were markedly reduced compared with the shNC group, while they were dramatically increased in the WTAP-OE group compared with the WTAP-NC group (**Figures 6A–C**, **7A–C**). Additionally, IHC analysis revealed an obvious reduction in the positive rate of both the WTAP and ki67 in the shWTAP plus cisplatin group compared with the other groups, while their expressions were obviously higher in the WTAP-OE plus cisplatin group compared with the WTAP-NC plus cisplatin group, and there was no obvious reduction compared with the WTAP-OE plus NS group (**Figures 6E**, **7E**).

Furthermore, we confirmed that the suppressed tumor growth in the shWTAP plus cisplatin group was attributed to the enhanced cell apoptosis, while the TUNEL assay indicated that the promoted tumor growth in the WTAP-overexpressing group was attributed to the decreased cell apoptosis (**Figures 6E**, **7E**). To further investigate the underlying mechanism, Western blotting analysis was conducted to examine the expressions of Bax and Mcl-1. The tumors from the shWTAP plus cisplatin group contained markedly higher levels of Bax as well as lower levels of Mcl-1 compared with the control group. By contrast, tumors from the WTAP-OE plus cisplatin group demonstrated lower levels of Bax but higher levels of Mcl-1 compared with the WTAP-NC plus cisplatin group, while there was no obvious change compared with the WTAP-OE plus NS group (**Figures 6D**, **7D**). Taken together, these results demonstrated that WTAP silencing could render chemo-sensitivity to cisplatin, while WTAP over-expression might promote the chemo-resistance to cisplatin in EC cells *in vivo*.

## Discussion

Chemotherapy treatment is a mainstay management for advanced and recurrent EC patients, while chemo-resistance still remains a challenge for successful treatment of such malignancy (26). Therefore, understanding the molecular mechanisms of chemo-resistance will be valuable for targeted EC treatment. WTAP, playing a critical role in transcriptional and post-transcriptional regulation, has been reported as an oncogene, its expression is elevated in multiple types of human tumors, and such up-regulation is associated with poor prognosis as well (9–17). To the best of our knowledge, the effect of WTAP on the resistance to cisplatin of EC cells has not yet been studied. Our current findings provided novel insights into the role of WTAP in the chemo-resistance to cisplatin of human EC cells.

In our current work, we assessed the expression of WTAP at the mRNA and protein levels in four EC cell lines. Because RL95-2 cells exhibited sufficient WTAP expression and ARK-2 cells were deficient in WTAP, RL95-2 and ARK-2 cells became ideal cell lines to test the functional role of WTAP. By depletion or over-expression of WTAP in RL95-2 or ARK-2 cells, respectively, we found that WTAP depletion decreased the cell proliferation and clonogenic survival properties as well as induced the cell apoptosis and cell cycle arrest in G2/M phase in response to cisplatin, suggesting that WTAP possessed vital effects on the sensitivity to cisplatin of human EC cells. Furthermore, *in vivo* data confirmed the involvement of WTAP in cisplatin sensitivity of EC cells. After treatment with cisplatin, WTAP-depleted EC cells formed smaller tumors in nude mice, while WTAP-overexpressing EC cells formed larger tumors compared with the corresponding control cells, respectively. Taken together, we demonstrated that WTAP could suppress chemo-sensitivity to cisplatin of human EC cells.

Studies have demonstrated that chemotherapy resistance is associated with the enhanced expression of WTAP in other cancer types (12, 18). For example, in pancreatic cancer (PC), WTAP can promote chemo-resistance of PC cells to gemcitabine through stabilizing Fak mRNA and activating Fak signaling pathways, including Fak-Src-GRB2-Erk1/2 and Fak-PI3K-AKT pathways (18). Fak inhibitor, GSK2256098, can reverse WTAP-induced tumor chemo-resistance to gemcitabine in PC. Moreover, WTAP depletion alone does not induce apoptosis of AML cells but significantly increases the extent of apoptosis compared with the control cells following getoposide treatment (12). These data imply that the increased WTAP expression is associated with the chemo-resistance to etoposide in AML. We, for the first time, demonstrated the promotive function of cisplatin resistance of WTAP. Nevertheless, the mechanism underlying the WTAP-induced EC cell resistance in response to cisplatin is not clearly defined.

Wnt signaling can activate β-catenin-dependent (also known as canonical) pathway and at least two well-characterized β-catenin-independent (also known as non-canonical) pathways, the Wnt/Ca2+ pathway and the planar cell polarity (PCP) pathway (27, 28). The intracellular level of β-catenin plays a key role in the canonical Wnt/β-catenin signaling (28). However, the Wnt/β-catenin pathway has been documented to be inhibited in WTAP-deficient cells by targeting the WTAP/WT1 complex (15, 29, 30). WT1 has been reported to interact with WTAP and act as a negative regulator of the Wnt signaling pathway (15, 29–31). Depletion of WTAP in CRC cells increases the binding activity of WT1 to induce the transducing β-like protein 1 (TBL1) expression, ultimately leading to the degradation of catenin and suppression of Wnt/β-catenin signaling (15). In brain arteriovenous malformation, lack of WTAP represses the Wnt/β-catenin pathway by enhancing WT1 activity, resulting in inhibition of angiogenesis of endothelial cells.

Consistent with previous studies, we observed that the high expression phenotype of WTAP was significantly correlated with the Wnt/β-catenin pathway according to the analysis of GSEA using the GEO database. This finding was further supported by the enhanced transcription of downstream apoptosis-associated genes (c-Myc, Surviving, and Bcl-xl) of the Wnt/β-catenin signaling pathway in the WTAP-overexpressing cells. Western blotting analysis and IF staining revealed that WTAP over-expression contributed to the cytoplasm-to-nucleus translocation of β-catenin, which was not observed in the WTAP-depleted cells. Besides, the level of GSK-3β was not changed due to WTAP over-expression. However, the phosphorylation level of GSK-3β at serine 9 was obviously enhanced. Wnt/β-catenin signaling is widely known to play a key role in multiple cellular functions, such as embryonic development, differentiation, adult tissue homeostasis, cell proliferation, cell motility, angiogenesis, and so on (32–35). In particular, aberrant activation of Wnt/β-catenin pathway has been correlated with tumorigenesis of EC, including accelerating proliferation of EC cells (36–38). These data suggested that WTAP could promote chemo-resistance to cisplatin through the Wnt/β-catenin pathway in EC.

In summary, our results showed that WTAP could accelerate chemo-resistance to cisplatin in EC cells through activating the Wnt/β-catenin pathway, which was further confirmed by our *in vivo* test. These results indicated that regulation of WTAP to suppress EC chemo-resistance to cisplatin might be clinically valuable, provide novel insights into the intervention of chemo-resistance to cisplatin in EC therapy. Further studies are still required to better elucidate the detailed mechanisms.

## Data Availability Statement

The original contributions presented in the study are included in the article/supplementary material. Further inquiries can be directed to the corresponding author.

## Ethics Statement

The animal study was reviewed and approved by Institutional Animal Care and Use Committee of Shandong Cancer Hospital and Institute.

## Author Contributions

WLX contributed to the study design and conducted the experiments. NL and XW participated in the study design. LW and WYX contributed to statistical analysis. XS supervised the research. All authors contributed to the article and approved the submitted version.

## Funding

This work was funded by the Taishan Scholars (no. ts201511073) and National Natural Science Foundation of China (NSFC 81672591).

## Conflict of Interest

The authors declare that the research was conducted in the absence of any commercial or financial relationships that could be construed as a potential conflict of interest.
